# *Marchantia polymorpha* Defense Against Snail Herbivory

**DOI:** 10.1002/pei3.70052

**Published:** 2025-04-14

**Authors:** Fabian Schweizer, Isabel Monte, Roberto Solano, Philippe Reymond

**Affiliations:** ^1^ Department of Plant Molecular Biology University of Lausanne Lausanne Switzerland; ^2^ Plant Molecular Genetics Department National Centre for Biotechnology (CNB), Consejo Superior de Investigaciones Cientificas (CSIC), Campus University Autonoma Madrid Spain

**Keywords:** gastropod, *Helix aspersa*, herbivory, liverwort, *Marchantia polymorpha*

## Abstract

During the course of evolution, higher plants have developed efficient strategies to cope with herbivory from arthropods. Upon perception of herbivore‐derived cues, the jasmonic acid (JA) signaling pathway is activated and triggers the expression of defense genes. The first land plants that arose ca. 500 Mya were bryophytes, including liverworts, and fossil records indicate that they were also exposed to herbivore pressure. Interestingly, recent studies showed that the liverwort *Marchantia polymorpha* contains a functional JA pathway that protects against insect feeding. However, since the appearance of insects is estimated to have occurred several million years after that of bryophytes, we hypothesized that this pathway could have been used to fend off contemporaneous gastropod feeders. Here, we challenged *M. polymorpha* with the land snail *Helix aspersa* and found that neonates grew significantly bigger on Mp*coi1*, a mutant in the JA pathway, than on wild‐type plants. This finding demonstrates that JA‐dependent defenses in a liverwort are effective against gastropod herbivory and suggests that this feeding group constitutes an additional selection pressure that may have arisen early during land plant evolution.

## Graphical abstract

Ancestral liverwort species were already equipped with the jasmonate pathway to fend off herbivores. However, insect appearance on earth does not fit with the estimated age of bryophytes, as they probably evolved million years later. We found that the snail *Helix aspersa* readily feeds on *Marchantia polymorpha* and that a Mp*coi1* mutant, where the jasmonate pathway is blocked, is significantly more susceptible to the snail. This novel finding suggests that gastropods may have contributed to the selection pressure exerted by herbivores on early land plants.

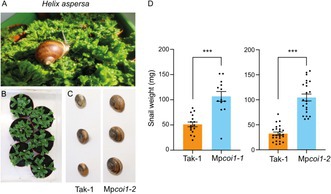

## 1 Introduction

Terrestrial colonization by plants has been a key evolutionary step in life history on Earth. It constituted the basis for a massive expansion of the green lineage, covering all continents and having a crucial impact on global climatic cycles, including CO_2_ fixation. On land, plants were rapidly exposed to novel abiotic and biotic stresses that constituted a strong selection pressure, leading to the emergence of adaptive traits but also to a coevolution process that has been postulated to explain the current abundance of plant species and their enemies. In particular, herbivory is considered a major driving force underlying this arms race, owing to the nutritious properties that plants provide to numerous types of feeding species (Ehrlich and Raven 1964; Després et al. 2007).

**FIGURE 1:**
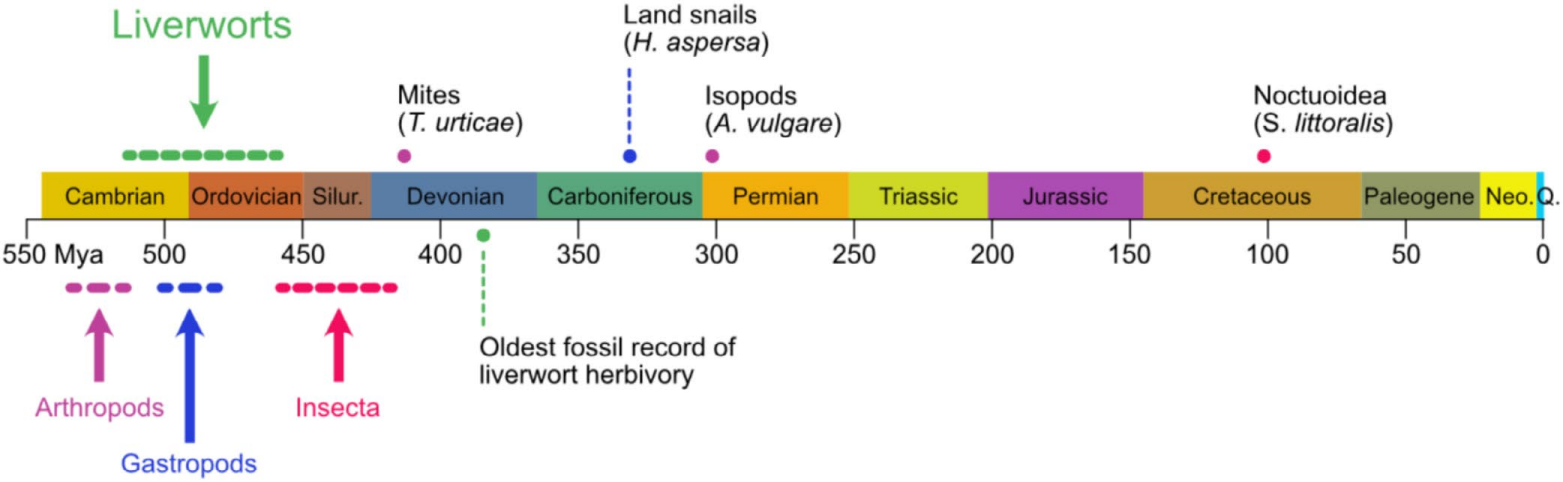
Estimated origin of liverworts and herbivores. Liverworts, to which the model bryophyte *Marchantia polymorpha* belongs, have appeared during the Cambrian‐Ordovician era. Yet, the first evidence of liverwort herbivory was documented in fossils from the mid‐Devonian. Founding liverwort species may have initially been exposed to herbivory from arthropods or gastropods, whose origin predates or coincides with that of liverworts. Herbivore species for which the role of JA‐dependent *M. polymorpha* defense has been demonstrated experimentally are indicated with the estimated origin of their group.

The first land plants were derived from ancestral freshwater algae and probably evolved into two major groups represented by extant tracheophytes (vascular plants) and bryophytes, which include the three lineages hornworts, liverworts, and mosses (Rensing 2018; Fürst‐Jansen et al. 2020). Although the order of appearance of the bryophyte lineages is not fully resolved, their origin has been situated in the Cambrian–Ordovician era, between 470 and 515 Mya, using fossil calibration and molecular clock methodology (Morris et al. 2018). However, another scenario based on fossil cryptospore records suggests an origin of the common ancestor of extant bryophytes at the more recent end of this range and places the first liverworts in the late Ordovician (Bowman 2022) (Figure 1).

Recently, the liverwort *Marchantia polymorpha* has become a useful bryophyte model to study land plant evolution, due to ease of growth and genetic manipulation, full genome sequence, and relatively minimal gene content with low redundancy in signaling pathways, including the jasmonic acid (JA) pathway (Bowman et al. 2017; Montgomery et al. 2020). Whether the ancestral liverworts were exposed to herbivory and developed resistance mechanisms is an important question that has recently attracted attention (Labandeira and Wappler 2023). Fossils represent a useful source of information about plant damage, even though bodies of the consumers might not have been preserved. The oldest evidence about liverwort herbivory comes from a study on preserved specimens of *Metzgeriothallus sharonae* in Middle Devonian siltstone deposits from the Hudson River Valley of eastern New York, USA (Hernick et al. 2008). Several examples of margin feeding, holes, and surface abrasion of thalli were found. In addition, signs of piercing‐sucking events were reported in the form of small punctures and scars (Labandeira et al. 2014). Although this report gives only indirect evidence of herbivory and cannot provide information on the precise herbivore group or species that fed on this liverwort, it supports the hypothesis that bryophytes were exposed to a broad‐spectrum herbivory.

Multiple examples of modern liverwort herbivory may also testify that these plants have since long been a host for various arthropods (Glime 2006; Hashimoto 2006; Colloff and Cairns 2011; Sawangproh and Cronberg 2016; Imada and Kato 2016). Strikingly, some lepidopteran insects were found to feed exclusively on the liverwort *Conocephalum conicum* , suggesting a potential adaptation to plant defenses (Imada et al. 2011). Flavonoids extracted from the liverwort *Marchantia linearis* showed significant insecticidal properties against *Spodoptera litura* , although whether this insect could feed on *M. linearis* was not tested (Krishnan and Murugan 2015). Interestingly, more than 100 years ago, a study postulated that oil bodies in liverworts contain deterrent compounds that are effective against land snails (Stahl 1888). Also, the observation that the arboreal land snail *Euhadra brandtii sapporo* feeds on mosses provides additional evidence that gastropods may have constituted a biotic pressure for liverworts. Indeed, the estimated origin of gastropods in the Cambrian–Ordovician era (Dinapoli and Klussmann‐Kolb 2010) coincides with that of the first land plants and, depending on the estimates, may have preceded the appearance of Insecta (Misof et al. 2014). Thus, the first land plants colonized an environment where non‐insect arthropods, already present in the Early Cambrian according to fossil records (Betts et al. 2014), and gastropods may have been potential attackers (Figure 1). However, there is not yet molecular evidence that bryophytes or liverworts defense is effective against a gastropod.

In vascular plants, herbivory triggers the induction of defenses that are regulated by the JA pathway. Upon recognition of herbivore‐associated molecular patterns by cell‐surface receptors, a signaling cascade leads to the expression of genes that encode defense proteins or enzymes responsible for the synthesis of toxic secondary metabolites (Erb and Reymond 2019). Signaling involves the release of linolenic acid from the chloroplast membrane and several biosynthetic steps, including the formation of precursors by allene oxide synthase (AOS) and 12‐oxophytodienoic (OPDA) reductase (OPR3), that lead to the cytosolic production of the bioactive hormone JA‐Ile. In the nucleus, JA‐Ile binds to a SCF^COI1^ complex and this triggers the degradation of JAZs, which are repressors of bHLH transcription factors MYC2, MYC3, MYC4, and MYC5. The release of transcriptional inhibition allows the activation of numerous defense genes (Browse 2009; Chini et al. 2016).

Recent groundbreaking work in *M. polymorpha* has revealed that the JA pathway was already functional in early land plants. It was, however, found that dinor‐OPDA was the ancestral ligand for COI1 (Mp2g26590) and that a single amino acid substitution in the COI1 ortholog from vascular plants allowed binding of the new ligand JA‐Ile (Monte et al. 2018). Nevertheless, a Mp*coi1‐1* mutant was impaired in wound‐induced gene expression and more susceptible to *Spodoptera littoralis* herbivory (Monte et al. 2018), illustrating the ancient defensive role of this pathway and mirroring the known role of Arabidopsis COI1 in defense against this lepidopteran herbivore (Schweizer et al. 2013). Similarly, a study on the only *M. polymorpha* MYC ortholog (MpVg00340) showed that this factor was necessary and sufficient to activate the JA pathway and a Mp*myc* mutant was, again, more susceptible to *S. littoralis* feeding (Peñuelas et al. 2019). In addition, insect‐induced accumulation of the sesquiterpenes thujopsene, β‐chamigrene, and cuparene, which are present in oil bodies, was abolished in Mp*myc* (Peñuelas et al. 2019). Interestingly, a recent study showed that a *M. polymorpha* mutant in the transcription factor MpC1HDZ (Mp3g02320) that controls oil body cell differentiation displayed a significant reduction in oil body numbers, a lower content of terpenoid‐related compounds, and was more susceptible to the isopod *Armadillidium vulgare* , a crustacean arthropod herbivore (Romani et al. 2020). This finding supports the role of the JA pathway in the production of anti‐herbivore terpenes in oil bodies but whether it is also important for oil body development should be investigated. Finally, CRISPR/Cas9‐mediated disruption of two allene oxide synthases in *M. polymorpha* , MpAOS1 (Mp3g21350), and MpAOS2 (Mp5g16260), led to a higher susceptibility to the spider mite *Tetranychus urticae*, highlighting a potential role of the JA pathway against ancient cell‐content feeders (Koeduka et al. 2022).

## 2 Results

**FIGURE 2:**
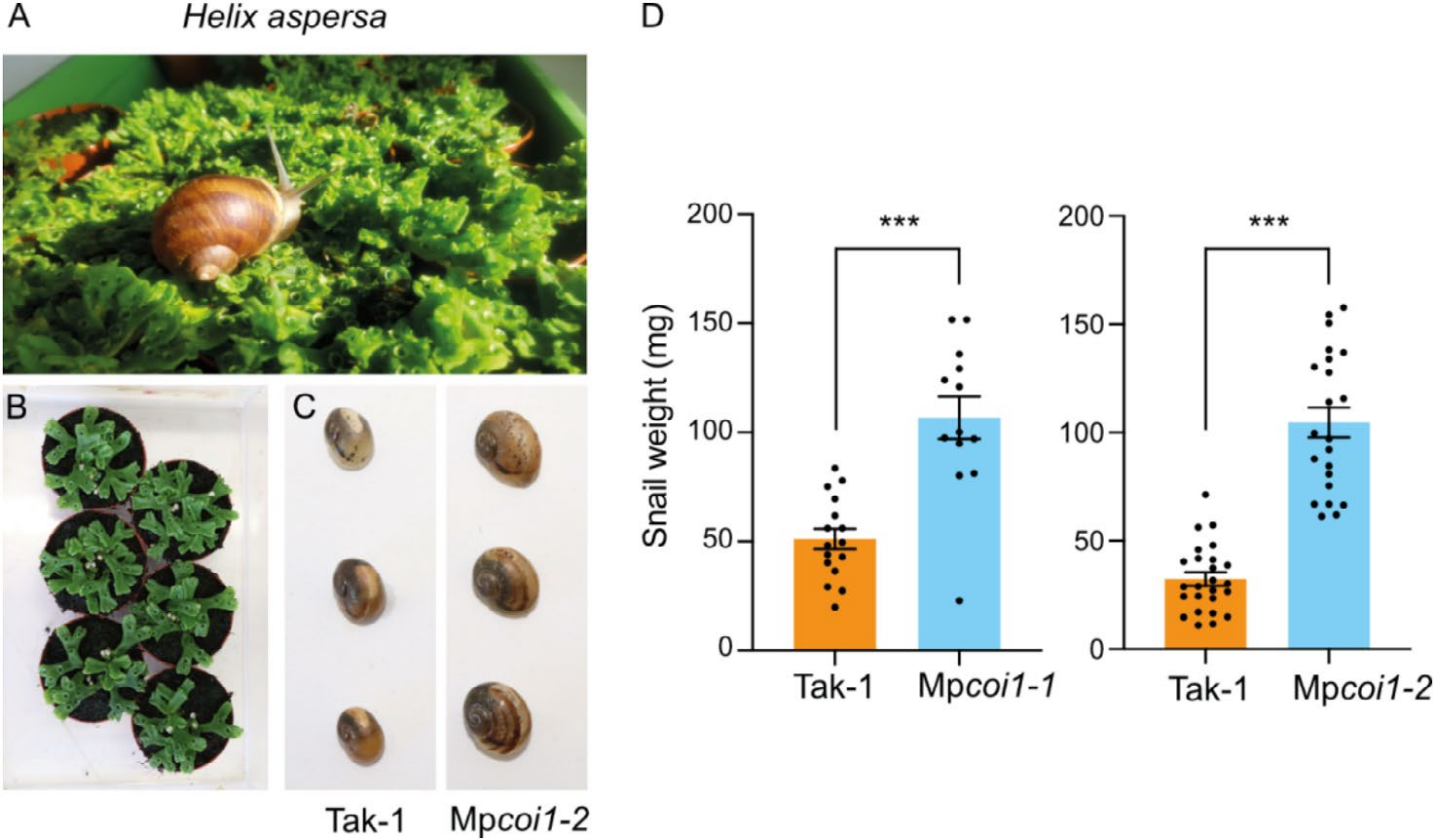
Snail performance on *Marchantia polymorpha* genotypes. (A) Adult *Helix aspersa* feeding on *M. polymorpha* . (B) *H. aspersa* neonates were placed on 5‐week‐old thalli for 14 days, after which pictures were taken (C) and snails were weighed (D). Mean ± SE is shown (*n* = 13–25 per genotype). Significant differences between genotypes are indicated (Student's *t*‐test, ***p < 0.001). Dots indicate individual values. This experiment was repeated with similar results.

In this study, we wanted to assess the contribution of the JA pathway in *M. polymorpha* defense against a gastropod. First, we reared the land snail *Helix aspersa* Müller (brown garden snail) and observed that adults readily consumed *M. polymorpha* thalli. Then, we challenged wild‐type or Mp*coi1* plants with *H. aspersa* neonates and measured snail development after 2 weeks. Strikingly, snails grew significantly bigger on the mutant alleles Mp*coi1‐1* and Mp*coi1‐2*, demonstrating that JA‐dependent defenses in a liverwort are effective against gastropod herbivory (Figure 2).

Collectively, experiments with extant species of arthropods and gastropods, fossil records, and studies with mutants of the JA pathway point to the crucial role of this pathway in early land plant defenses against a broad range of herbivores. However, the precise contribution of each group and the exact nature of ancestral herbivore species that fed on liverworts are difficult to assess. Indeed, the first record of liverwort herbivory was found on specimens that lived ca. 100 Myr after the apparition of the first bryophytes (Figure 1) and the lack of herbivore preservation in fossils may hinder the identification of more ancient liverwort‐herbivore interactions. Anyhow, the current study unveils a novel role for the JA pathway and allows considering gastropods as an additional selection pressure that may have arisen early during land plant evolution. Whether the same defensive secondary metabolites play a role against different types of herbivores is an interesting question that will deserve further investigation. Also, the possibility that the JA pathway is crucial for defense in the sister mosses and hornworts lineages is another tempting hypothesis. However, given that oil bodies are restricted to liverworts, the nature of anti‐herbivore compounds in these lineages might be different.

In addition to the finding of fungal hyphae in fossilized *M. sharonae* (Hernick et al. 2008), there are reports that *M. polymorpha* can also be infected by the oomycete *Phytophthora palmivora* (Carella et al. 2018) and by naturally occurring pathogenic fungal strains, including the vascular wilt *Fusarium oxysporum* and the necrotroph *Irpex lacteus* (Matsui et al. 2020; Redkar et al. 2022). Strikingly, Mp*coi1‐2* was more susceptible to *I. lacteus* infection, suggesting that the JA pathway may also have been crucial for resistance against ancestral necrotrophs (Matsui et al. 2020).

Although recent studies, including this work, illustrate the power of *M. polymorpha* as one model system to reconstruct the evolution of plant biotic interactions, inference about the defense system of the last common ancestor of land plants is complicated by the probable early split between tracheophytes and bryophytes, accompanied by a lack of whole genome duplication in the latter group (Bowman et al. 2017). Anyhow, in the future, deeper molecular analyses should focus on how components of the JA pathway, some of which are already present in green algae (Wang et al. 2015), were gradually assembled to fend off pathogens and herbivores.

## 3 Materials and Methods

*M. polymorpha* accession Takaragaike‐1 (Tak‐1; male) was the wild‐type. Generation of Mp*coi1*‐*1* and Mp*coi1*‐*2* knockout lines has been described elsewhere (Monte et al. 2018). *Helix aspersa* Müller was obtained from Gurmels, Switzerland (https://schneckenpark.ch). Snails were reared in a plexiglass box (20°C) in turf‐containing soil and fed with sweet potato. Snails were kept in a moist environment by water spraying once a day. Eggs were collected and placed in closed styrofoam boxes for 2–3 weeks until hatching.

For snail herbivory bioassays, *M. polymorpha* gemmae were grown on half Gamborg's medium (Duchefa) containing 1% agar in continuous light (20°C, 120 μmol m^−2^ s ^−1^) for 7 days. Thalli were then transferred to soil (three per pot) and grown for 4 weeks (21°C, 10/14 h light/dark cycles, 100 μmol m^−2^ s^−1^) in a transparent plastic box to maintain high humidity. Then, 20–25 neonate *H. aspersa* were placed on thirty 5‐week‐old thalli. After 10 to 14 days of feeding, snails were collected and weighed using a precision balance (Mettler‐Toledo XP205).

## Conflicts of Interest

The authors declare no conflicts of interest.

## Data Availability

The data obtained in the study are presented in the article.
